# Biophysics of Tumor Microenvironment and Cancer Metastasis - A Mini Review

**DOI:** 10.1016/j.csbj.2018.07.003

**Published:** 2018-07-27

**Authors:** Bashar Emon, Jessica Bauer, Yasna Jain, Barbara Jung, Taher Saif

**Affiliations:** a Mechanical Science and Engineering, University of Illinois at Urbana-Champaign, United States; b Division of Gastroenterology and Hepatology, Department of Medicine, University of Illinois at Chicago, United States; c Department of Architecture, BRAC University, Dhaka; d Bioengineering, University of Illinois at Urbana-Champaign, United States

**Keywords:** Tumor biophysics, Cancer, Metastasis, ECM stiffness, Growth factors, Activin/TGFβ, ECM, Extracellular matrix, CAF, Cancer associated fibroblast, LOX, Lysyl Oxidase, IGFs, Insulin-like growth factors, EGF, Epidermal growth factor, TGFβ, Transforming growth factor β, VEGF, Vascular endothelial growth factor, MMPs, Matrix metalloproteinases, KGF, Keratinocyte growth factor, also FGF7, IL-6, Interleukin-6, IL-33, Interleukin-33, IL-13, Interleukin-13, HGF/SF, Hepatocyte growth factor/Scatter factor, FGF, Fibroblast growth factor, CTGF, Connective tissue growth factor, ADAMs, Adamalysins, EMT, Epithelial to mesenchymal transition, TACs, Tumor-associated collagen signatures, CSF-1, Colony stimulating factor 1, SDF-1/CXCL12, Stromal cell-derived factor 1/C-X-C motif chemokine 12, TNF-α, Tumor necrosis factor-α, NO, Nitric oxide, α-SMA, α-Smooth muscle actin, ANGPT2, Angiopoietin 2, CYR61/CCN1, Cysteine-rich angiogenic inducer 61/CCN family member 1

## Abstract

The role of tumor microenvironment in cancer progression is gaining significant attention. It is realized that cancer cells and the corresponding stroma co-evolve with time. Cancer cells recruit and transform the stromal cells, which in turn remodel the extra cellular matrix of the stroma. This complex interaction between the stroma and the cancer cells results in a dynamic feed-forward/feed-back loop with biochemical and biophysical cues that assist metastatic transition of the cancer cells. Although biochemistry has long been studied for the understanding of cancer progression, biophysical signaling is emerging as a critical paradigm determining cancer metastasis. In this mini review, we discuss the role of one of the biophysical cues, mostly the mechanical stiffness of tumor microenvironment, in cancer progression and its clinical implications.

## Graphical abstract

**Figure:**
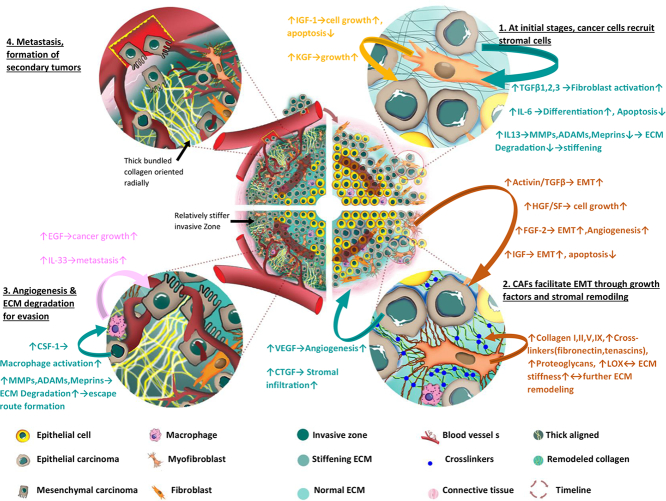

## 1 Introduction

Despite significant improvement in both early diagnosis and treatment of cancer patients, metastasis is still the major cause of mortality. It is responsible for 90% of about 500,000 cancer deaths each year in the United States [1]. Cancer transformation and metastasis are driven by both genomic changes in the tumor cells and the architecture and environmental context of the host and target tissue or organ [2, 3]. In addition, the process is subjected to various signals such as growth factors, cytokines, chemotactic stimuli and extracellular matrix modifications. Accordingly, cancer progression is often conceptualized as a continuum in which a cell changes over time from a benign phase into an invasive, metastatic phenotype as it responds to various cues from the microenvironment along the way. While this metamorphosis clearly requires activation and inactivation of specific genes, it is recognized that this process also involves changes in the biophysical phenotype of the cells and tissue, such as the adhesive force mechanics responsible for both cell-cell and cell-extracellular matrix (ECM) interactions [4]. The interplay between the biophysical properties of the cells and ECM establishes a dynamic reciprocity between neoplastic cells and tumor stroma consisting of immune and inflammatory cells, fibroblasts, capillaries, and the ECM scaffold [[5], [6], [7]]. This dynamic reciprocity appears to regulate a wide range of cellular responses critical to tumorigenesis, including initiation of metastasis. The growing list of players in the biophysical interactions contains matrix stiffness, pore size, viscoelasticity, crosslinking proteins and density, fiber network configuration, cancer cell stiffness [[8], [9], [10], [11], [12], [13], [14]]. Cells sense, process, and respond to mechanical and other biophysical cues from the ECM using a coordinated mechanochemical system composed of adhesion receptors, cytoskeletal networks, and molecular motors [[15], [16], [17], [18], [19], [20], [21], [22]]. The mechanism by which these mechanotransduction events takes place is very complex and diverse. In some cases, individual molecules are responsible when cellular tension in response to ECM rigidity exposes a cryptic signaling molecule or unfolds a propeptide chain; in some other cases, the strain in the cyto-structure can regulate receptor-ligand interaction to affect enzyme activity or, control a mechanosensitive ion channel [17, [23], [24], [25]]. Also, other physical parameters such as change in nuclear volume and shape [26, 27], cell membrane curvature [28, 29], fluid permeation [30], plasticity [31, 32] etc. play significant roles in different scenarios where physical cues are converted into biochemical responses at a cellular level. On a tissue level, solid stress [[33], [34], [35], [36]], interstitial fluid pressure [[37], [38], [39], [40]] and topographic features are a few of many mediators that influence various stages of disease developments, especially neoplasia, fibrosis, cancer etc. Understanding how the mechanical microenvironment regulates cancer cell biological processes responsible for metastasis represents a newly developing paradigm. This new paradigm has the potential to add novel anti-metastasis therapeutics to the current arsenal. This review will focus particularly on stiffness characteristics of tumor microenvironment at various stages, its relationship with metastatic progression and its significance in potential clinical applications for improved diagnosis and treatment.

## 2 Evolution of differential stiffness and pro-metastatic architecture within tumor stroma

**Fig. 1:**
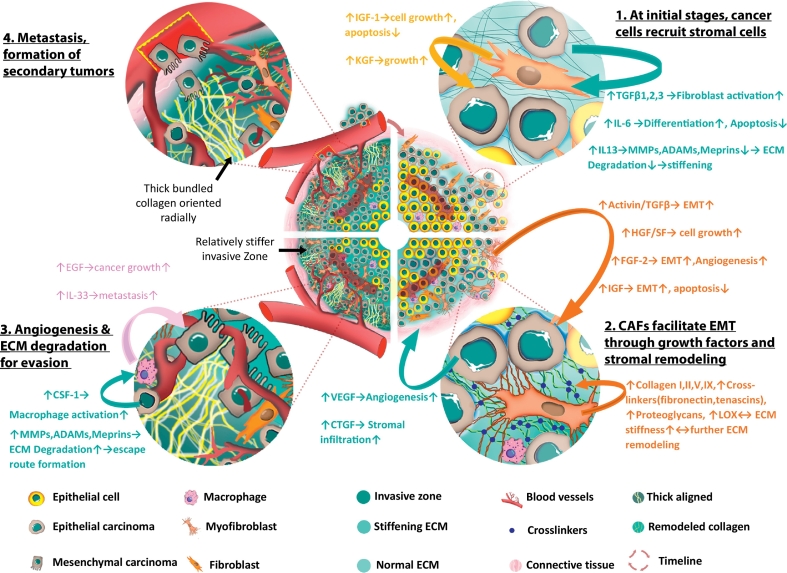
Chronological development of pro-metastatic stromal architecture and the major factors involved. Segment 1 (top right): At the early stages of cancer, epithelial cancer cells secrete various growth factors that facilitates fibroblast activation, differentiation, downregulates ECM degradation by reducing MMPs and hence increase stiffness. In response, stromal fibroblasts regulate factors such as IGF, KGF etc. that promote cell growth and inhibit apoptosis. Segment 2 (bottom right): Upon activation, fibroblasts manifests myofibroblast (or, CAF) signatures and produce activin/TGFβ, IGF that stimulate EMT; HGF that increases cell growth; FGF-2 that increases angiogenesis and so on. In addition, CAFs continue to remodel and reinforce ECM by depositing collagen I, II, V, IX, increasing crosslinking, upregulating LOX and thus stromal stiffness gradually goes up. Due to excessive cellular proliferation and tumor growth, a region at the core becomes hypoxic and cancer cells increase secretion of VEGF and CTGF that are known to support angiogenesis and infiltration respectively. Segment 3 (bottom left): As carcinoma cells go through EMT, they produce CSF-1 which activates macrophages that in turn produce EGF, IL-33 etc. that promotes metastasis. At some regions of the invasive front, the stromal cells align thick collagen bundles radially that can be used as an escape route by the metastatic cells. Eventually, aggressive cancer cells degrade stiff ECM by upregulating MMPs, ADAMs etc., evade stroma, infiltrate lymph nodes and blood vessels and go through metastasis. Segment 4 (top left): Migrating cancer cells anchor at distant sites and starts the process all over to develop secondary tumors. [[51], [58], [66], [67], [68], [69], [70], [71]].

For most breast and colorectal cancer types, stiffness of neoplastic tumors is considerably higher than neighboring normal tissue [[41], [42], [43]] and is considered to be highly correlated with cancer progression and metastasis [44, 45]. Although the definite role of the augmenting stiffness in cancer progression still remains enigmatic [44, 46], current knowledge suggests that gradual stiffening of tumor stroma can primarily be attributed to deposition and remodeling of ECM [12, 44, [47], [48], [49]]. The mechanism of stromal transformation is complex, and it encompasses a myriad of chemical and physical agents and processes. Cancer cells, cancer associated fibroblasts (CAFs) and macrophages work in concert to modulate ECM within the tumor microenvironment through the following activities:

a) excessive deposition of structural components such as collagen I [45], collagens II,III,V,IX [[50], [51], [52]], cross-linker glycoproteins (fibronectin, tenascins etc.) [[53], [54], [55]], proteoglycans (heparan sulphate, CD44) [56, 57],
b) secretion/regulation of various growth factors and cytokines e.g. IGF1, EGF, TGFβ, VEGF etc. [58, 59], ECM-transforming enzymes e.g. matrix metalloproteinases (MMPs) [60, 61], lysyl oxidase (LOX) [[62], [63], [64]], transglutaminase [51, 65] and.
c) orchestrating topographic reconfiguration of the stroma, such as alignment of ECM fibers, amid the plethora of aforementioned activities. Fig. 1 presents a succinct illustration of the process.

Both spatial and temporal variations of such soft to semi-rigid transformation of cancerous tumors is of clinical significance. In cases of colorectal and breast cancer, it is observed that collagen I, LOX expression, and consequently stiffness, is significantly upregulated at later stages (III/IV) compared to early stages (I/II) [43, 45, 72]. Moreover, the fact that increased stiffness is associated with advanced stages of various carcinoma and potential metastasis, makes stiffness a promising prognostic and/or therapeutic target. In fact, not only stiffness, its spatial distribution within the neoplasm and accompanying manifestations such as indistinct stromal boundary [72], collagen fiber characteristics (length, width, waviness etc.) [72], tumor-associated collagen signatures-1,2,3 (TACS 1–3) i.e. local densification, fibril straightening/stretching, radial alignment near boundary [73, 74] are also under extensive scrutiny as probable markers of tumor progression, metastasis and patient survival [45, 74]. For example, it was found in a recent study comparing grade I/II to grade III canine mammary gland carcinomas, that grade III cases are more likely to exhibit thicker, longer and straighter collagen fibers and less likely to have a defined tumor-stromal boundary [72]. Again, within the stroma of numerous solid tumors, comparatively denser regions of collagen (i.e. TACS-1) are found to be co-localized with aggressive phenotypes of cancer cells [73, 75, 76]. Another fascinating feature of spatial distribution of stiffness, as reported by Acerbi et al. [43], is that tissue stiffness in the invasive region of the stroma is significantly higher than that in tumor core or adjacent normal tissue. Also, increased mechanical heterogeneity within tumor was found to positively correlate with more aggressive subtypes of human breast cancer. Furthermore, a remarkable architectural metamorphosis transpires in parallel with tumor progression. Straightened collagen fibers aligned normal to the boundary (TACS-3) congregate to form distinct bundles in parts of peripheral regions [74]. These bundles of remodeled stiff collagens might pave the way for the cancer cells, as invasion ‘highways’ [46], to escape and metastasize to secondary sites. Hence, such transformation of tumor morphology can turn out to be very crucial and must be addressed going forward.

## 3 Stromal metamorphosis and metastasis: a story of reciprocity

Metastasis is a very important juncture in cancer progression and long has been considered as the principal therapeutic target [77]. Contemporary understanding, especially for breast and colon cancer development, is that metastatic progression goes hand in hand with tumor micro-environmental transition i.e. from softer tissue to stiff fibrous state. The ‘context’ [2] of tumor development is multifaceted in such a convoluted manner that most of the events are dynamically involved in a feed-forward loop rather than a cascade. We will discuss the cycle of events around tumor stiffness and topography.

### 3.1 ECM stiffness

#### 3.1.1 Effect of ECM stiffness on cancer cells and other stromal inhabitants

**Fig. 2:**
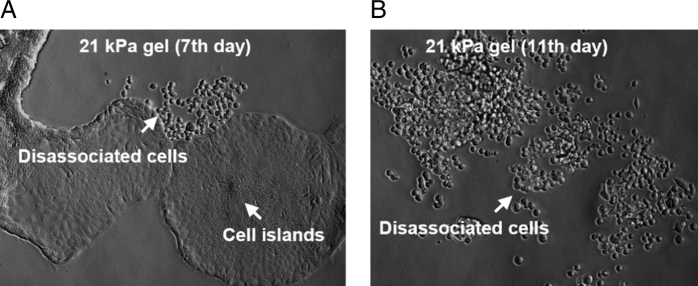
Epithelial cancer cells, perhaps, wait for stromal stiffness to reach an optimum level before they decide for EMT. HCT-8 cells (human colon cancer) adhere to 21 kPa polyacrylamide (PA) gel substrate, functionalized with fibronectin, and form cell islands. On substrate of appropriate rigidity (here 21 kPa), they dissociate from each other and become rounded after 7 days of culture (a) [93]. Within few more days, most of the cell islands become R cells. The R cells are more tumorigenic in mouse models, and express several oncogenes [92].

Extra-cellular matrix stiffness has a considerable influence on cellular behavior. Increased stiffness, mostly through mechanotransduction, may elicit a wide range of responses from different types of cells. For example, breast epithelial cells in a stiff 3D matrix become more proliferative following high Rho activity, FAK phosphorylation and adhesion [78]. Again, apart from autocrine/paracrine TGFβ and stromal cell-derived factor-1 (SDF-1 or CXCL12) [79] signaling, ECM stiffness plays an important role in differentiation of stromal fibroblasts into cancer associated fibroblasts (CAFs). P190B RhoGAP overexpression in association with elevated extra-cellular and cytoskeletal tension activates latent TGFβ that, in turn, activates fibroblasts [80]. Interestingly, ECM stiffness of ∼16 kPa [81], which is typical Young's modulus of fibrotic/cancer tissue (1.08–68 kPa) rather than normal tissue (0.38–7.33 kPa) [82], was found to be the threshold for upregulated expression of α-SMA, a proven myofibroblast (also CAF) marker [81, 83]. Also, a transcription factor which facilitates CAF generation and maintenance is YAP/TAZ, which requires high stiffness and actomyosin contractility for activation [84]. For epithelial cells, Provenzano et al. [85] suggested that high-stiffness matrix is necessary for maintaining invasive phenotype and culpable for upregulation of a set of cancer-associated genes dubbed as ‘proliferation signatures’ [52, 86]. Even the immune cells such as macrophages are sensitive to surrounding rigidity. Macrophages grown in-vitro on high stiffness substrates yield more pro-inflammatory mediators (e.g. TNF-α, NO, IL-1β etc.) than macrophages on softer substrates [87]. Intriguingly, all these responses by these cells are, in a sense, self-induced. It is the cells themselves that synthesize and remodel ECM to change its composition, arrangement and rigidity. In response, ECM prompts the cells to adapt to altered environment by changing their behavior and activities. Very often, this change in cellular activities leads to even more modulation of the matrix and stroma. Thus, a reciprocal exchange between the stromal cells and ECM results in a dynamically adaptive cycle that contributes fatefully towards malignant tumor progression. Numerous research suggest that the mechanical microenvironment of tumor stroma gradually shifts towards a metastatic niche. ECM stiffness is one of the key elements of stromal biophysics and increasing evidence is corroborating the fact. For example, a stiffer microenvironment, induced by increased collagen crosslinking in breast cancer tumors in vivo, promotes initiation of metastasis [12, 49]. An appropriately stiff fibrin gel microenvironment produces a metastatic variant of murine B16-F1 melanoma cells that are highly tumorigenic in animal models [88]. Cellular actomyosin activity and force generation depend on the stiffness of the microenvironment [[89], [90], [91]]. Thus tumor mechanical microenvironments may influence metastatic transition through local force cues that generate or select a subset of metastatic cells [92]. And different cell types may keep tuning their surrounding environment until a suitable stiffness is attained. For instance, our previous work shows that HCT-116 and DU-145 cells (colon and prostate cancer cells respectively) express metastatic pheno- and geno-types on 10 kPa polyacrylamide (PA) gels whereas HCT-8 cells (colon carcinoma cells) express similar phenotype on 21 kPa substrates [92, 93]. These epithelial type cells (*E*-cells) become rounded (R-cells) when cultured on appropriately stiff substrates for about 7 days (Fig. 2). The R-cells are more tumorigenic in mouse models compared to their E counterparts. They express several oncogenes and suppress apoptotic genes. None of the above mentioned cells manifested similar transformations on very low stiffness substrates (1 kPa PA gels) or very high stiffness substrates (3.6 GPa polystyrene) [92]. Moreover, these metastatic cells tend to be softer than the normal cells [92]. Thus the role that ECM stiffness plays in metastatic progression is quite significant.

#### 3.1.2 Biophysics of activin/TGFβ signaling in metastasis

Cancer genome sequencing confirmed key genes whose mutations can drive tumorigenesis [94] and have solidified components of the TGFβ superfamily as drivers of pathogenesis in colon cancer. These include inactivating mutations in the TGFβII receptor (TGFBR2), the activin receptor 2A (ACVR2A) and downstream signaling target SMAD4 [94]. TGFβ and activin are involved in the regulation of cell proliferation, differentiation, migration and apoptosis [[95], [96], [97]]. Activation of SMAD2/3/4 proteins through ligand binding in the canonical pathway leads to translocation to the nucleus and transcriptional regulation of target genes to affect growth suppression and upregulation. The non-canonical pathway is SMAD4-independent and engages other signaling pathways [95, 96]. Activin and TGFβ both have dual and opposing roles in colon carcinogenesis as they may promote growth suppression, as well as migration and metastasis in more advanced colon cancer, also known as the molecular switch [[98], [99], [100], [101]]. In early stage colon cancer, the TGFβ super family is growth suppressive, while in advanced disease, high serum and stroma levels of TGFβ are associated with poor prognosis in colon cancer [102, 103] and in pancreatic cancers [104].

**Fig. 3:**
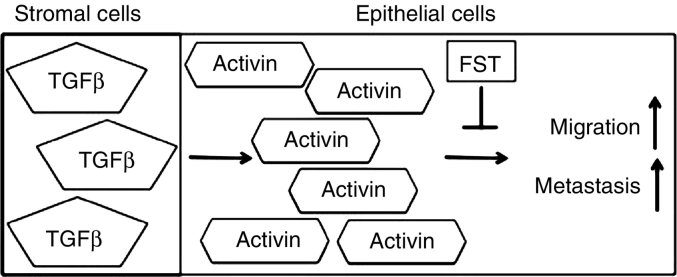
TGFβ from the stromal cells increases the activin ligand in epithelial cells and in serum which is required for an increase in epithelial cell migration leading to metastasis. This process can by blocked by the activin specific ligand trap, follistatin.

Our published data indicate that TGFβ induces activin secretion from colon tumor stromal cells which acts to promote metastatic behavior in epithelial cells as measured by increased cell migration and increased epithelial to mesenchymal transition (EMT) [105]. Normal colorectal fibroblast and epithelial colon cancer cell line were analyzed for activin ligand expression following TGFβ stimulation. After TGFβ treatment, activin secretion was increased in colon cancer epithelial cell irrespective of active SMAD4. Interestingly, levels of activin secretion both at baseline and after TGFβ treatment were substantially higher either in stromal cells alone or in co-cultures of stromal with epithelial cells, indicating that the stroma is a significant source of secreted activin. Transwell migration assay with colon cancer cells showed that both activin and TGFβ individually increase cell migration. Treatment with activin specific inhibitor follistatin (FST) that inhibit activin signaling but not TGFβ, confirms that TGFβ induced cell migration is dependent on activin signaling while FST does not inhibit TGFβ induced growth suppression (Fig. 3) [105].

In addition, activin signaling is a key element in metastatic pancreatic cancer as evidenced by elevated serum activin levels in pancreatic cancer patients with poor prognosis [104]. Also, over-expression of activin in mouse xenografts of pancreatic cancer cells led to larger tumors and significantly decreased weight indicative of tumor cachexia [104]. Ohnishi et al. [106] showed that activin induces collagen secretion from pancreatic stellate cells in a dose dependent manner and described the role of activin in the development of pancreatic fibrosis. We speculate that activin release from the stromal cells might be related to the ECM stiffness around them. Our preliminary experiments support this possibility. We plated colon cancer associated fibroblasts on 2, 10 and 40 kPa gel substrate, mimicking various stiffness microenvironment and observed that greater stiffness of the stroma increased the activin level in the epithelial cells.

Due to immense significance of metastasis in disease development, elucidating the forces driving metastasis is critical for developing interventions in cancer progression. An important event underlying metastasis is EMT. Extensive literature has established links between transcriptional factors (EMT-TFs) such as Snail1 [107, 108], ZEB [109], Twist [110, 111] and metastatic processes of cancer cells e.g. E-cadherin downregulation, angiogenesis, and intravasation [107]. A review by Wei and Yang [112] sheds light on various mechanotransduction pathways that integrate physical cues from tissue rigidity and biochemical signaling to drive cancer cell plasticity and metastatic promotion. Recent developments indicate that EMT-TFs are also expressed in CAFs and are instrumental in coordinated plasticity progression, increased proliferation and chemoresistance [113, 114]. ECM rigidity, again, is found to be influential in this process. For instance, increased ECM stiffness induces ROCK activity through elevated intracellular tension, integrin accumulation and signaling to ERK2 which stabilizes Snail1 to nucleus [115]. By regulation of Snail1, CAFs within stiff matrix can control YAP1 level and activities [115]. Elevated YAP interaction with TEAD/TEF transcription factors enhances growth, transformation, migration, and invasion [116]. Expression in CAFs of another transcription protein, Twist1, also correlates with tumor growth, invasion depth and lymph node metastasis [117]. Remarkably, both Twist1-activated fibroblasts and Snail1-expressing CAFs stimulate ECM stiffness [118], which provides yet another evidence of feed-forward operation. Furthermore, there is evidence suggesting that macrophages aid collagen synthesis by organizing collagen I into fibrillar bundles [119], even though they are not known for collagen production. In addition, macrophages infiltrate in higher numbers into invasive lesions of higher stiffness and their density correlates with increased cellular TGFβ signaling at the invasive front, corroborated by evidence from human breast cancer biopsies [43]. Thus, tissue mechanics and inflammation may cooperate to drive aggressive progression in tumor.

#### 3.1.3 Effect of ECM stiffness on endothelial cells

**Fig. 4:**
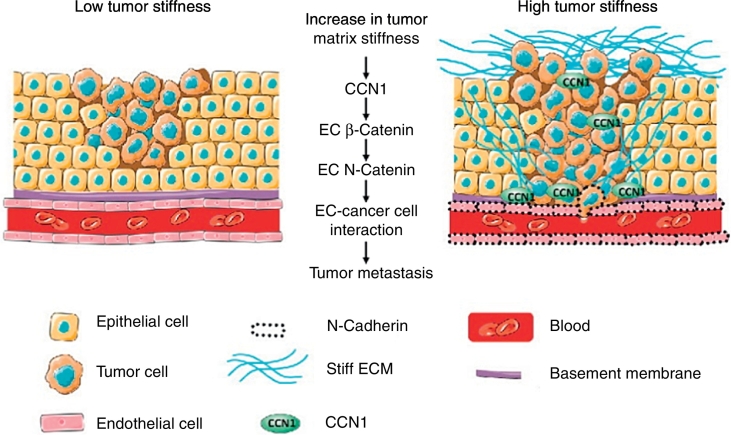
Influence of tumor stiffness in endothelial CCN1(CYR61) facilitated metastasis [127].

Endothelial cells (ECs) are important in cancer progression, however, the influence of tumor stiffness on the endothelium is largely unknown. With newly developing device systems, we are learning more about how endothelial cells create a vascular network within tumor stroma and facilitate nutrients and oxygen supplies to the hypoxic core where solid stress as well as interstitial fluid pressure is high [[120], [121], [122]]. In addition, ECs secrete a number of “angiocrine factors” such as ANGPT2, FGF, IGF, IL, CSF, SDF1 etc. that promote metastatic progression [123, 124]. Also, ECs play their part in ECM remodeling for establishing vascular niches in the stroma. The EC basement membrane comprises various ECM constituents e.g. laminin α4 (LAMA4), fibronectin, hyaluronan and collagen-α type IV etc. and acts as a storage of various cytokines and growth factors [124]. Another noteworthy protein, CCN1 (CYR61) is a matricellular protein that is known to be involved in a number of tumorigenic processes e.g. cell adhesion, migration, fibrosis, apoptosis, angiogenesis etc. [125]. A recent study on endothelial cells found that expression of CCN1 is higher on stiffer substrates (in-vitro) and also stiffer regions of orthotopically transplanted tumors (in-vivo) [126]. This stiffness-induced CCN1 is considered to be upregulating N-cadherin on endothelium through activation of β-catenin nuclear translocation and signaling [126, 127]. N-cadherin, in turn, facilitates interaction between cancer and endothelial cells and facilitates aggressive cells' (transitioned to mesenchymal state) infiltration into blood/lymph vessels for metastasis (see Fig. 4).

### 3.2 Cancer cell stiffness and stromal morphology

Cancer cells, irrespective of the type of carcinoma, are usually softer than their normal counterparts [128, 129]. This softening of neoplastic cells has several implications. Perhaps, one of the reasons why cells undergo such a transformation is preparing for breaking out of the primary site through the stromal ECM scaffold. There are a few modes of their evasion such as amoeboid migration, mesenchymal migration, and collective invasion. Single cell or amoeboid migration is somewhat independent of ECM breakdown and cells usually maneuver their way through tissue gaps and trails by alteration of their shapes. Mesenchymal cells, in contrast, utilize FAK, Rho activities and proteolytic ECM degradation to ‘force’ their way out adopting a spindle-like morphology. In collective migration, some mesenchymal cells lead the way for a strand of cells that follow the traction pull. Overall, all of these migration mechanisms are influenced by both cancer cell stiffness and architectural properties e.g. ECM dimension, density or gap size, orientation, stiffness [46, 75, 130, 131].

## 4 Dual agents in cancer microenvironment

One fascinating aspect of cancer microenvironment that adds to the complexity of tumor progression is the dual role played by several agents, both physical and chemical. The dual agents can act as both cancer suppressor and promoter and choose their course of action based on the signals received from the surroundings. Thus, such actors can be potential therapeutic targets that can be manipulated to reverse the effects of cancer progression. A quintessential example of such a dual agent is activin/TGFβ. In preceding sections, we have discussed how TGFβ expression in stromal fibroblasts and carcinoma cells depends on and/or influences stromal stiffness to promote metastatic progression. In contrast, TGFβ signaling can also act to suppress tumor progression through regulation of cell growth, apoptosis and immortalization [132]. Thus, during tumor progression, various genomic coding for protective and cytostatic TGFβ signaling is either mutated or deleted. As a result, TGFβ signaling switches to promote cancer progression, invasion, and tumor metastasis [132]. Activin A has also demonstrated both oncogenic and tumor suppressor roles. In prostate and breast cancer it proved to be a tumor suppressive actor while in lung, head and neck squamous cell carcinoma, its expression is correlated with increased growth, invasion and poor patient prognosis [95]. Although the interaction between activin signaling and tumor microenvironment is not well understood, recent findings indicate that activin A signaling operates in a cell-type and context dependent manner. For instance, it can exert positive functions such as cell cycle arrest in non-invasive cells, but can increase proliferation in aggressive cancer cells [95, 133]. Thus, activin expression in the tumor may provoke differential outcomes. During tumor growth, stromal cells like fibroblasts secrete activin A to inhibit growth, but cancer cells can adapt and TGFβ stimulates EMT by regulating transcription factors such as ZEB, Snail or Twist. In addition, TGFβ has been shown to stimulate collective migration primarily through extracellular-regulated kinase 1/2 (ERK1/2) activation [95, 134, 135]. We have evidence that on substrates of higher stiffness, fibroblasts exert more force and also produce more activin/TGFβ. Thus gradual remodeling and stiffening of stromal ECM appears to be an important contributing factor for role switching of TGFβ.

The classic double agent in cancer biophysics is the extracellular matrix (ECM). As a mediator between biomechanics and tumor biology, ECM can play a suppressor role at early stages of tumor progression; but at later stages, it can radically change its role and convert to a promoter of invasion and metastasis. Fang et al. discussed inhibiting and promoting activities of collagen at different stages of cancer development and how it can behave like a ‘double-edged sword’ in tumor progression [46]. Traditionally, ECM has been considered as a physical scaffold that binds cells and tissues together. In cancer, collagen was regarded as a steric hindrance to cell motility and invasion and also biochemically inert. However, recent findings show that ECM can also elicit biochemical and biophysical signaling [48, 49] that may modulate cell adhesion, migration, angiogenesis, tissue remodeling and metastasis in cancer. ECM remodeling through increased deposition of collagen, protease-dependent and –independent cross-linking and stiffening, proteolytic degradation etc. are relevant to metastatic processes at various stages of tumor development. One of the key factors in ECM remodeling is Lysyl oxidase (LOX) which is an enzyme that catalyzes the cross-linking of collagens or elastin in the ECM and thus regulates the tensile strength of tissues. However, recent results revealed additional activities of LOX such as gene transcription, motility/migration, and cell adhesion [136]. Due to its influence on such diverse functions, LOX also can play multiple roles in cancer. Several studies found that LOX (*ras* mRNA) is a potent tumor suppressor gene in some stromal cells that happened to be inhibiting signaling pathways that induce carcinogenic transformations [[136], [137], [138]]. For instance, Giampuzzi and colleagues demonstrated that LOX-downregulated normal kidney fibroblasts led to increased cellular proliferation and anchorage-independent growth, loss of PDGF and IGF-1 regulation, and constitutive activation of *ras* [139]. Although tumor suppressive characteristics of LOX is still to be explored for better understanding, its contribution in promoting cancer is much more pronounced. Numerous research has demonstrated a positive correlation between LOX expression and migration [140, 141], invasion, EMT [[142], [143], [144]], metastasis [142], and poor patient prognosis. In summary, tumor microenvironment is a complex realm where interaction between various physical and chemical aspects are abundant. There are plenty of agents other than TGFβ, LOX or collagen that exhibit such dual nature and thus are potential therapeutic target. Harnessing any of these agents within tumor microenvironment may lead to novel treatment methods in cancer.

## 5 Translational relevance

Increased matrix stiffness not only promotes tumor progression by enhancing metastasis, but also impedes transport of therapeutic agents reducing the efficacy of chemotherapy which is a critical problem for the delivery of treatment. In cases of canine breast cancer, poor prognosis was associated with increased tumor stiffness and a tumor associated collagen signature (TACS) of increased collagen density, fiber width, length and straightness [72]. The clinical use of elastography allows assessment of tissue stiffness and could provide a biomarker for more aggressive disease. Marangon et al. [145] utilized shear wave elastography (SWE) in combination with mild hyperthermia and thermal ablation to treat a murine model of epidermoid carcinoma. They noted an initial transient increase in tumor stiffness followed by a softening of the tumor with subsequent treatment leading to a reduced tumor volume when compared to untreated tumor-bearing mice which has the potential to translate into a new therapeutic approach. Heat treatment of hepatocellular carcinoma (HCC) cell lines was used by Zhang et al. [146] to mimic radiofrequency ablation therapy. The heat treated HCC were combined with synthetic matrix of a spectrum of stiffness and implanted as xenografts. They observed that insufficient heat treatment led to significant promotion of HCC proliferation and increased stiffness enhance cell motility. Importantly treatment with vitamin K1 combined with Sorafenib reverses the effects of increased stiffness indicating the potential for cancer therapies which reduce tumor stiffness.

## 6 Summary

In this mini review we discussed the current state of understanding on the role of tumor stiffness on cancer progression. The detailed molecular mechanism by which stiffness influences the dynamic interactions between cancer cells and the stroma is only beginning to emerge. However, it is becoming evident that tumor biophysics may offer a new paradigm for understanding cancer and for novel therapeutics. And with the advancement of micro- and nano-tool technologies, we are breaking new barriers and beginning to understand better how closely physics and biological processes are linked. As a result, we are seeing an increase in the effort to understand tumor biophysics better and potentially employ physical and mechanical characteristics as diagnostic, prognosis or therapeutic purposes. For future, a vast avenue is yet to be explored in this research direction. Our current understanding of various biochemical signaling in cancer is mostly based on 2D cell culture and thus, developing methods to study these pathways in an ex-vivo 3D platform can help us better understand how exactly these physical parameters affect chemical signaling. Also, since stromal stiffening is related with poor prognosis, can we revert tumor remodeling to prevent metastasis? Or, can we use tumor rigidity or solid stress as indicators of cancer progression and patients' outcome? Moreover, although we know ECM stiffness influences activin secretion, we still do not know how exactly cells convert this physical cue into a biochemical signal and control growth factor synthesis and release. Such questions are currently being addressed by various laboratories around the world. Insights from these studies are expected to add new arsenal against cancer.
